# The Major Chromophore Arising from Glucose Degradation and Oxidative Stress Occurrence during Lens Proteins Glycation Induced by Glucose

**DOI:** 10.3390/molecules23010006

**Published:** 2017-12-22

**Authors:** Felipe Ávila, Guillermo Schmeda-Hirschmann, Eduardo Silva

**Affiliations:** 1 Escuela de Nutrición y Dietética, Facultad de Ciencias de la Salud, Universidad de Talca, Talca 3460000, Chile; 2 Instituto de Química de los Recursos Naturales, Universidad de Talca, Talca 3460000, Chile; schmeda@utalca.cl; 3 Laboratorio de Química Biológica, Facultad de Química, Pontificia Universidad Católica de Chile, Santiago 7820436, Chile; esilva@uc.cl

**Keywords:** glucose, oxidative stress, cataract disease, eye lens proteins, protein glycation

## Abstract

Glucose autoxidation has been proposed as a key reaction associated with deleterious effects induced by hyperglycemia in the eye lens. Little is known about chromophores generated during glucose autoxidation. In this study, we analyzed the effect of oxidative and dicarbonyl stress in the generation of a major chromophore arising from glucose degradation (GDC) and its association with oxidative damage in lens proteins. Glucose (5 mM) was incubated with H_2_O_2_ (0.5–5 mM), Cu^2+^ (5–50 μM), glyoxal (0.5–5 mM) or methylglyoxal (0.5–5 mM) at pH 7.4, 5% O_2_, 37 °C, from 0 to 30 days. GDC concentration increased with incubation time, as well as when incubated in the presence of H_2_O_2_ and/or Cu^2+^, which were effective even at the lowest concentrations. Dicarbonylic compounds did not increase the levels of GDC during incubations. ^1^H, ^13^C and FT-IR spectra from the purified fraction containing the chromophore (detected by UV/vis spectroscopy) showed oxidation products of glucose, including gluconic acid. Lens proteins solutions (10 mg/mL) incubated with glucose (30 mM) presented increased levels of carboxymethyl-lysine and hydrogen peroxide that were associated with GDC increase. Our results suggest a possible use of GDC as a marker of autoxidative reactions occurring during lens proteins glycation induced by glucose.

## 1. Introduction

**Scheme 1:**
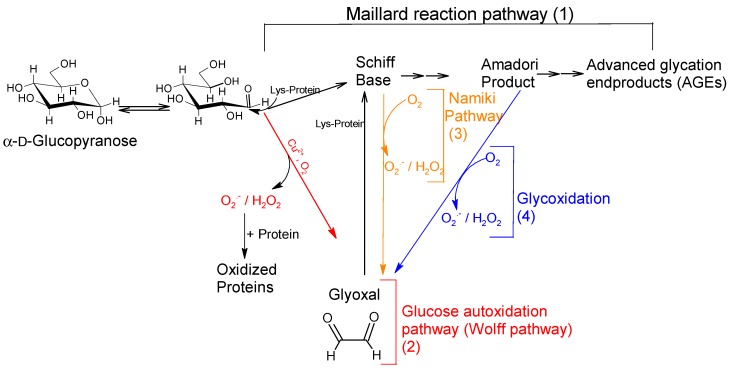
Pathways to induce non-enzymatic post-translational modifications mediated by glucose in proteins.

Sustained hyperglycemia states constitute a risk factor for the development of numerous non-communicable chronic diseases, including cataract disease [1]. Hyperglycemia (states) has been associated with the generation of advanced glycation end products (AGEs), which in crystallins proteins of the human eye lens have been shown to increase during aging and also in nuclear cataract disease [2]. Glucose can induce post-translational modifications in proteins by several pathways, including: (1) direct Maillard reaction, which occurs between the carbonyl group (open-chain form) and nucleophilic amino acid residues; (2) glucose autoxidation (Wolff pathway) and posterior Maillard reaction; and (3) oxidative reactions by means of reactive oxygen species generation that can arise from glucose autoxidation and/or Namiki and glycoxidative pathways (Scheme 1). Glucose autoxidation has been proposed as an important pathway to generate reactive dicarbonyl compounds and AGEs during protein glycation [3]. The analysis of incubations performed in vitro with glucose and lysozyme or RGD-α3NC1domain of collagen 1 has shown that the glucose autoxidation products, glyoxal and methylglyoxal, were responsible for inhibiting the enzymatic and receptor–ligand interaction, respectively [4]. On the other hand, ex vivo analysis of AGEs in eye lens proteins from human cataractous lenses has shown that an Amadori product derived from the reaction between glucose and Lys or methylglyoxal was the most abundant modification [5]. This fact underscores the importance of understanding the contribution of autoxidative degradation pathways of glucose to the generation of AGEs as well as determining the effects of these reactions in protein function and structure. We have reported that glucose degradation under simulated eye lens physiological conditions can generate a single chromophore that possesses photosensitizing activity [6,7]. Photosensitizing reactions induced by this chromophore have previously shown to induce protein oxidation, crosslinking and cytotoxic effects in eye lens epithelial cells [6,7]. However, the chemical pathways that generate this chromophore as well as its chemical structure are unknown. The occurrence of the different reactions shown in Scheme 1 in the eye lens proteins are favored due to the extremely long lifetime of crystalline proteins, and a decrease in the levels of antioxidants such as ascorbate or glutathione occurring during eye lens aging and in cataract disease [8,9,10]. This pro-oxidant environment has been proposed to play a key role in the occurrence of oxidative post-translational modifications occurring in eye lens proteins, which can result in the generation of chromophores, fluorophores, protein crosslinking, aggregation and fragmentation [11,12]. However, the synergic effect between oxidative stress and glucose degradation has been poorly studied. In this work, the effect of pro-oxidant conditions and dicarbonilic stress, both related with the etiology of cataract disease, were studied in terms of the generation of the major chromophore arising from glucose degradation. The association between oxidative stress and glycoxidative modifications occurring during eye lens proteins glycation by glucose was assessed to determine a possible use of GDC as a marker of autoxidative reactions. 

## 2. Results

### 2.1. Effect of Oxidative and Glycoxidative Stress in the Generation of the Major Chromophore Arising from Glucose Degradation

**Figure 1:**
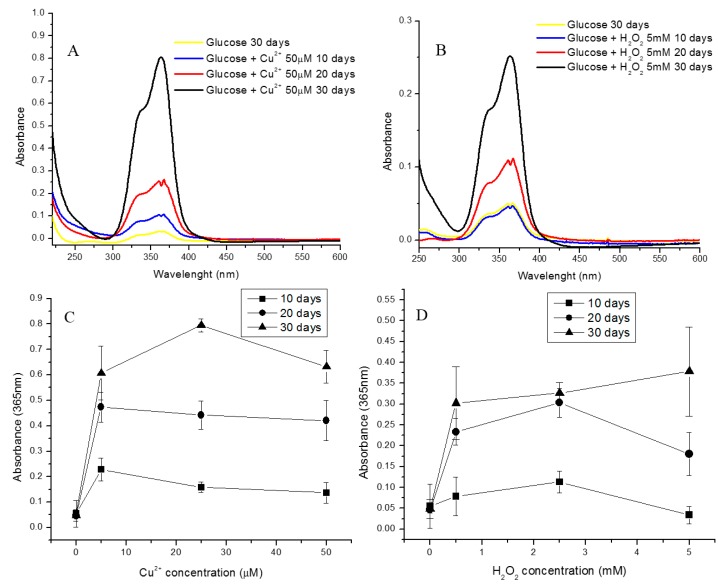

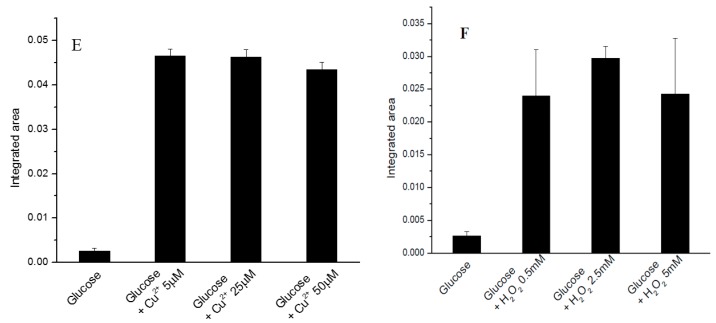
Effect of H_2_O_2_ and Cu^2+^ in the generation of the major chromophore arising from glucose degradation. Glucose (5 mM) was incubated during 10, 20 and 30 days at pH 7.4, 37 °C with different concentrations of Cu^2+^ or H_2_O_2_. Panels (**A**,**B**) show absorption spectra of incubated samples; Panels (**C**,**D**) show the absorbance determined at 365 nm, changing the concentration of Cu^2+^ or H_2_O_2_; Panels (**E**,**F**) show the area under the curve analysis, integrating the absorbance at 365 nm by each concentration along incubation time. Error bars indicate the standard deviation of the mean.

**Figure 2:**
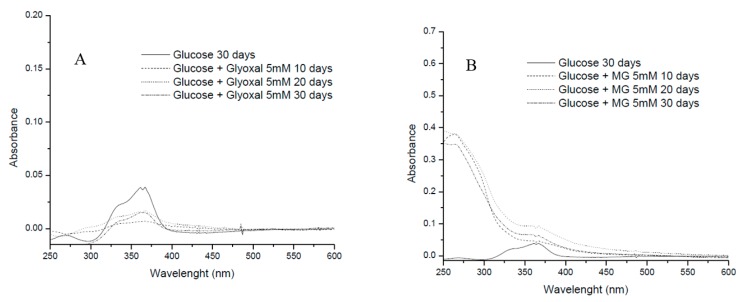
Effect of glyoxal and methylglyoxal in the generation of the major chromophore arising from glucose degradation. Glucose 5 mM was incubated during 10, 20 and 30 days at pH 7.4, 37 °C with glyoxal (5 mM) or methylglyoxal (5 mM). Panels (**A**,**B**) show absorption spectra of incubated samples during 10, 20 and 30 days of incubation at pH 7.4 and 37 °C.

**Figure 3:**
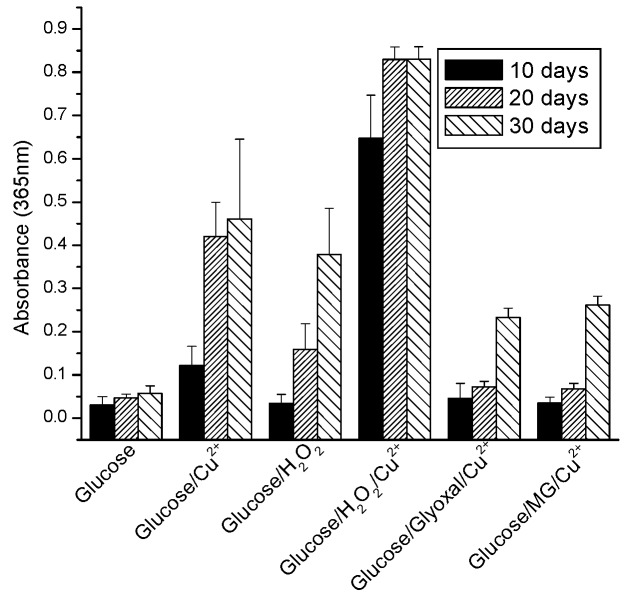
Effect of mixtures: glucose (5 mM)/Cu^2+^ (5 μM), glucose (5mM)/H_2_O_2_ (0.5 mM)_,_ glucose (5 mM)/Cu^2+^ (5 μM)/H_2_O_2_ (0.5 mM), glucose (5 mM)/Cu^2+^ (5 μM)/glyoxal (0.5 mM) and glucose (5 mM)/Cu^2+^ (5 μM)/methylglyoxal (5 mM) in the generation of the major chromophore arising from glucose degradation. Incubations were performed during 10, 20 and 30 days at pH 7.4 and 37 °C. Error bars indicate the standard deviation of the mean.

The effect of oxidative stress in the generation of the major chromophore arising from glucose degradation was analyzed by means of incubation of glucose (5 mM) with Cu^2+^ (5–50 μM) or hydrogen peroxide (0.5–5 mM) during 10, 20 and 30 days at pH 7.4 and 37 °C. Figure 1A,B shows the absorption spectra of chromophores generated during glucose degradation as a consequence of 10, 20 and 30 days of incubation in the presence of Cu^2+^ (5 µM) and H_2_O_2_ (5 mM), respectively. It can be observed that similar absorption spectra were obtained, independent of the pro-oxidant agent used in the incubations (Cu^2+^ and H_2_O_2_). Figure 1C,D shows the absorbance (at 365 nm) generated during 10, 20 and 30 days of incubation in the presence of increasing concentrations of Cu^2+^ (5–50 μM) or hydrogen peroxide (0.5–5 mM), respectively. It can be observed that both pro-oxidant agents induced an increase in the generation of the chromophores that possess an absorption maximum at 365 nm even at the lowest concentration used in these experiments. The increase in the absorbance was concomitant with the incubation time, which indicates an accumulation of the chromophores generated during the different times (Figure 1C,D). Figure 1E,F shows the area under the curve for the time profile plots for the generation of the chromophore at 365 nm, for each concentration. It can be observed that Cu^2+^ produced an increase in the levels of the glucose degradation chromophore (GDC) of 17.8 times when compared with glucose controls (Figure 1E). This increase reached a plateau at the lowest concentration used in these experiments (5 μM). Hydrogen peroxide at the lowest concentration (0.5 mM) induced an increase of 9.3 times in the levels of GDC when compared with glucose controls. Figure 2A,B shows that glyoxal and methylglyoxal did not generate the chromophore with the characteristic absorption maxima at 365 nm. Figure 3 shows that incubations carried out with both H_2_O_2_ and Cu^2+^ possessed a synergic effect in the generation of the major chromophore arising from glucose degradation. This effect was not observed when incubations were performed with mixtures glyoxal/Cu^2+^ or methylglyoxal/Cu^2+^ (Figure 3).

### 2.2. Glucose Oxidized Derivatives Are Present in Enriched Fractions Containing the Major Chromophore Arising from Glucose Degradation

**Figure 4:**
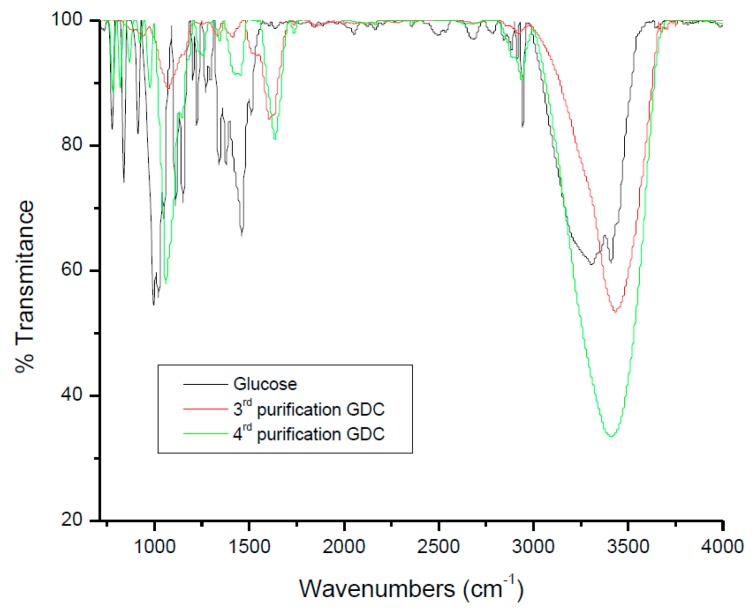
FT-IR spectra of glucose and enriched fractions of the glucose derived chromophore (GDC) purified three and four times consecutively by means of Sephadex LH-20.

Glucose 250 mM was incubated with Cu^2+^ 5 μM during 30 days at pH 7.4 and the resulting solutions were then purified by means of column chromatography. Figure 4 shows the FT-IR analysis of glucose and GDC. FT-IR (KBr) of glucose showed signals at: 3415, 3305 (OH, broad, s), 2949, 2915, 1520, 1468 (m), 1346, 1228, 1149, 1118, 1205 (s), 993 (s), 840 cm^−1^ in agreement with the NIST Chemistry WebBook [13]. In the FT-IR of GDC enriched fractions, the intensity of the OH band at 3436 cm^−1^ (OH, broad, s) was stronger than in glucose. The absorption at 1630 (m) suggests the occurrence of a carbonyl function or C–O stretching vibration. The lower absorption wavelength of the C=O stretching vibration can be explained by the influence of hydrogen bonding. The absorbance at 1066 cm^−1^ can be related to the C–O stretching vibrations.

**Figure 5:**
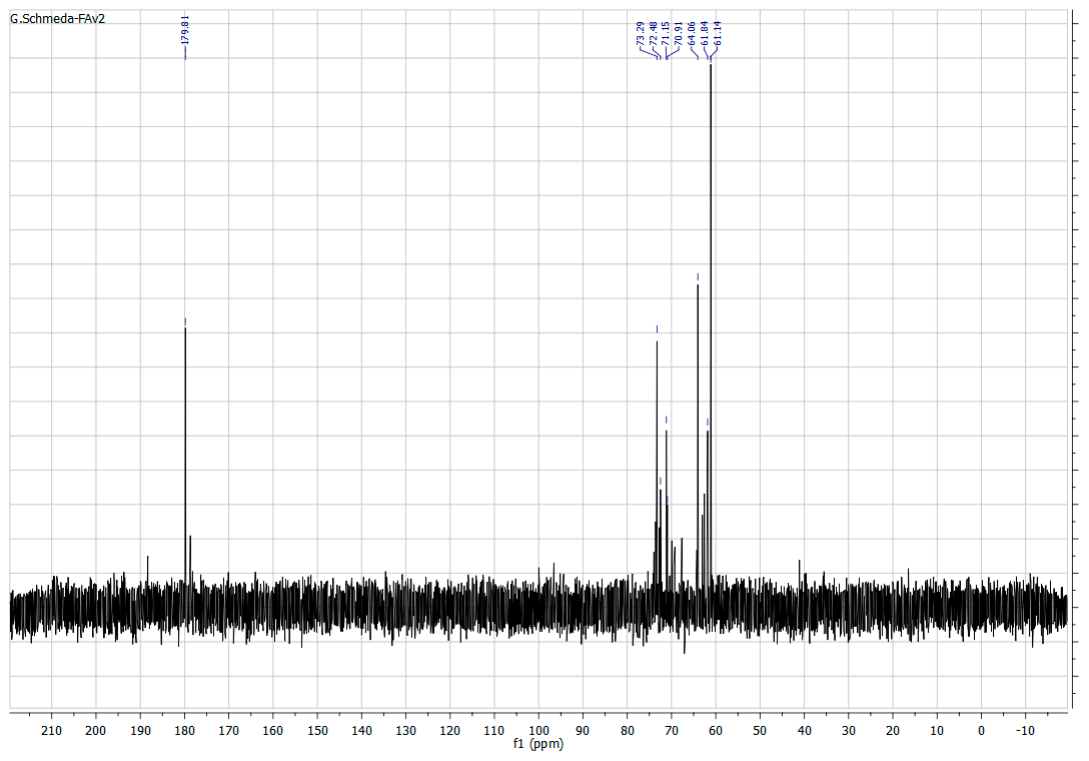
^13^C-NMR spectrum of the fraction showing the chromophore in UV/vis. While neither aromatic C nor conjugated double bonds are detected, the spectrum showed the oxidation product of glucose.

In the ^1^H-NMR of the compound, a broad group of overlapping signals appeared in the range δ 3.4–4.2 ppm. No hemiacetal H were observed. Literature data for the ^13^C-NMR spectra of the α- and β-anomer of glucose show the typical hemiacetal C at δ 92.7 and 96.5 ppm, respectively, while C2, C3, C4, C5 and C6 appear at 72.14, 73.4, 70.4, 72.10 and 61.3 ppm for the α-anomer and 74.8, 76.4, 70.3, 76.6 and 61.5 for the β-anomer, respectively [14]. The ^13^C-NMR spectrum of GDC showed a carbonyl function at δ 179.81 ppm, assignable to a carboxylic acid or ester (lactone) and doublet (d) at δ 73.29, 72.48, 71.16 and 70.92 ppm for H–C–OH atoms as well as a triplet (t) at δ 64.06 (minor) and 61.14 ppm (main) (–CH_2_OH) (Figure 5). The presence of a carboxylic acid function suggests that the product is gluconic acid, originated by oxidation of the glucose C-1. The occurrence of two t for the C-6 signal (CH_2_OH) indicates an equilibrium between the lactone (gluconolactone) and the open-chain form of gluconic acid. Furthermore, methylation of the product with diazomethane yielded a mixture with minor compounds showing two q at δ 54.99 and 55.05 ppm (methyl ester; δ 3.76 and 3.73 ppm in the ^1^H-NMR spectrum) and additional carbonyl C at δ 178.72 and 178.24 ppm, in agreement with possible isomers of the free carboxylic acid function. Hemiacetal signals and double bond sp^2^ carbons were absent, showing that the sugar is not a polyhydroxyaldehyde or an unsaturated sugar [15].

**Figure 6:**
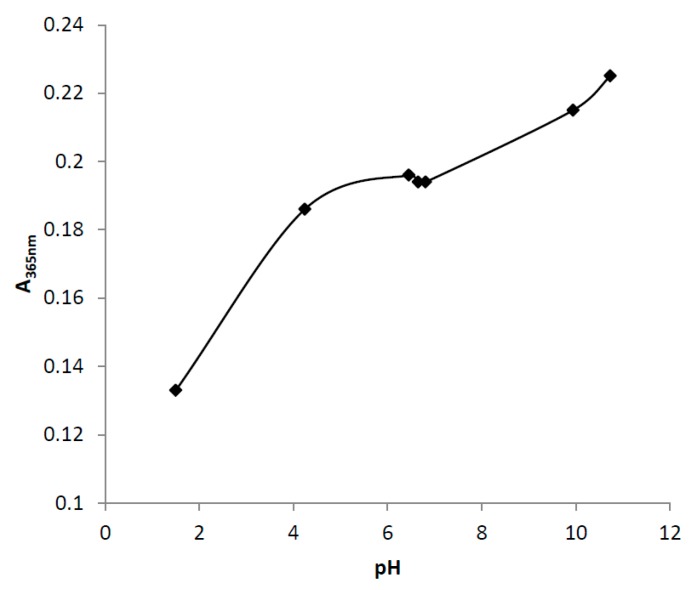
Changes in the absorption at 365 nm of enriched fractions of the major chromophore arising from glucose degradation (1.3 mg/mL) with pH.

The results above suggest that GDC could be a coordination complex of a carboxylic acid arising from glucose oxidation and Cu^2+^. With the aim to determine the effect of the pH in the absorbance of GDC, solutions at different pH were prepared and a constant volume of GDC was added in order to achieve a constant concentration of 1.3 mg/mL. Figure 6 shows that the absorbance of GDC depends on the pH. It can be observed that at pH 1.5 the absorbance reaches its minimum value, which corresponds approximately to the half of the absorbance values when compared with pH 10.73. The absorbance increased until reaching approximately constant values at pH 6.45–6.81 and a slight increase in the absorbance at basic pH was observed (Figure 6).

### 2.3. The Major Chromophore Arising from Glucose Degradation Generation Is Associated with Oxidative and Glycoxidative Modifications in Lens Proteins during Protein Glycation

**Figure 7:**
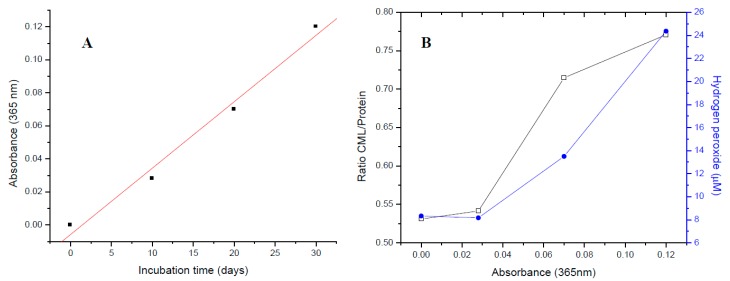
Relationship between the major chromophore arising from glucose degradation and markers of oxidative stress during eye lens protein glycation by glucose. Bovine eye lens proteins (10 mg/mL) were incubated during 10, 20 and 30 days in sterile conditions with glucose 30 mM and Cu^2+^ 5 µM. Panel (**A**) shows the correlation between the absorbance at 365 nm and the incubation time; Panel (**B**) shows the change in the absorbance at 365 nm for samples protein/glucose/Cu^2+^, previously deproteinized with carboxymethyl lysine levels and hydrogen peroxide. Symbols (□) indicate the ratio between the areas obtained from densitometric analysis of Western blots detecting carboxymethyl-lysine and the area obtained by means of blue coomassie staining. (●) Indicate the hydrogen peroxide concentration generated in the mixture quantified by means of the FOX method.

The association between the generation of the major chromophore arising from glucose degradation with oxidative stress and glycoxidative modifications in lens proteins was assessed incubating glucose (30 mM) with eye lens proteins (10 mg/mL) in the presence of Cu^2+^ (5 μM) during 10, 20 and 30 days at pH 7.4 and 37 °C. The absorbance at 365 nm was measured after lens proteins precipitation with trichloroacetic acid, and increased linearly with the incubation time (Figure 7A). Pearson correlation coefficients between GDC (r = 0.9925, *p* = 0.0076), hydrogen peroxide (r = 0.9833, *p* = 0.0167) and carboxymethyl-lysine levels (r = 0.9484, *p* = 0.0516) were calculated with incubation time, only correlations involving GDC and hydrogen peroxide were significant (*p* < 0.05). Figure 7B shows the association between the absorbance of the chromophores arising from glucose degradation at 365 nm with the levels of hydrogen peroxide and carboxymethyl-lysine generated by the incubation of the samples lens proteins/glucose/Cu^2+^during 10, 20 and 30 days. It can be observed that the levels of hydrogen peroxide and carboxymethyl lysine (CML) increased after 20 days of incubation (Figure 7B). We did not observe any increase of protein hydroperoxides after incubation of the mixture lens proteins/glucose/Cu^2+^ for 10, 20 and 30 days. When hydrogen peroxide was determined for samples glucose/Cu^2+^, higher levels were observed in comparison with the samples incubated in the presence of proteins, generating 30.0 ± 4.6, 49.6 ± 3.5 and 36.3 ± 4.4 μM for samples incubated during 10, 20 and 30 days, respectively. Linear correlations between the absorbance at 365 nm and levels of hydrogen peroxide and CML were established. Pearson correlation coefficients calculated for hydrogen peroxide (r = 0.9589, *p* = 0.0412) and CML (r = 0.9520, *p* = 0.048) were statistically significant (*p* < 0.05).

## 3. Discussion

Non-enzymatic reactions mediated by glucose have been proposed to play an important role in the etiology of nuclear cataract disease [16]. The analysis of post-translational modifications in human eye lens has shown evidence indicating a central role of glucose in the generation of advanced glycation end products in this tissue [5]. An association between conditions linked to the etiology of nuclear cataract such as increased levels of glucose, oxidative stress and increased levels of dicarbonylic compounds have been reported [17,18,19,20]. In particular, it has been determined that glucose in the presence of metals can be oxidized to generate reactive dicarbonylic species such as glucosone or glyoxal [3,21]. In this work we demonstrated that a synergism of deleterious effects produced by reactions between pro-oxidants and glucose resulted in the generation of the major chromophore arising from glucose degradation (GDC). This compound could be of interest because it easily detectable by means of UV-visible spectroscopy and could also constitute a marker of autoxidative reactions occurring in biological systems.

We have recently determined that glucose degradation mediated by copper traces can give rise to a single chromophore, which possesses photosensitizing activity, inducing oxidative reactions in eye lens proteins and bovine lens epithelial cells [6,7]. To determine the effect of pro-oxidant agents in the generation of GDC, glucose was incubated with Cu^2+^ or hydrogen peroxide (Figure 1). Cu^2+^ traces have been reported to catalyze glucose autoxidation and it has been reported that hydrogen peroxide is a by-product of glucose autoxidation [22]. We have found here that both Cu^2+^ and hydrogen peroxide could induce the generation of the major chromophore arising from glucose degradation (Figure 1). In our experiments, constant levels of GDC with Cu^2+^ or H_2_O_2_ concentrations were observed. A similar behavior has been found in glucose oxidation under alkaline conditions, which has shown an oxidation rate of zero-order in Cu^2+^, with enolization as the rate limiting step [23]. In agreement with this fact, we have found that GDC was produced even at the lowest concentrations of Cu^2+^ (5 μM) and H_2_O_2_ (0.5 mM) (Figure 1). This fact is relevant considering that physiological levels of hydrogen peroxide detected in vivo in aqueous humor from cataractous lenses are present in a range from 0.01 to 0.66 mM [24]. Incubations in the presence of the mixture glucose/Cu^2+^/H_2_O_2_ showed an increase in the generation of GDC (Figure 3). These results indicate that GDC was generated under oxidative stress conditions, possibly involving hydroxyl radical-mediated reactions. This possibility agrees with our results showing that dicarbonylic stress induced by glyoxal and methylglyoxal did not induce the generation of GDC (Figure 2).

With the aim to determine whether oxidative modifications are present in the chemical structure of GDC, NMR and FT-IR experiments were performed with purified samples. The ^13^C analysis of purified fractions containing GDC showed a signal at 179.8 ppm (Figure 5), which could be attributed to a carbonyl group resonance. ^1^H-NMR spectra also indicated the presence of oxidative modifications, showing signals that could correspond to aldehyde or lactone groups. These results agree with the FT-IR experiments that showed a band with a maximum at 1637 cm^−1^ for GDC purified four times, which could be attributed to a carbonyl stretching vibration (Figure 4). A similar band, showing a maximum at 1639 cm^−1^, has also been observed in a FT-IR analysis of glyoxal [25]. These experiments demonstrate that oxidative modifications are present in purified fractions containing GDC and agree with our results that showed an increase in GDC levels by Cu^2+^ and H_2_O_2_.

The fact that gluconic acid was found in enriched fractions of GDC can give rise to the generation of Cu^2+^ complexes, considering that our system contains low amounts of this metal. It has been reported that copper complexes can be formed by the interaction between the metal and two carboxylic acid groups, giving rise to an increase in the absorbance in the UVA-visible region, which has shown to be pH dependent [26,27]. For this reason, the change in the absorbance of GDC at 365 nm with the pH was assessed. At pH 1.5 the absorbance showed the lowest value, this could be attributed to the lowest amount of Cu^2+^-GDC complex, which occurs by coordination with the carboxylate oxygen of GDC. The subsequent increase in the GDC absorbance with the pH can be attributed to an increase in the carboxylate concentration and therefore an increase of the Cu^2+^-GDC complex. These results are in agreement with previous reports showing complexes of copper-carboxylic acid of numerous molecules, including gallic acid [28] and nicotinic aromatic carboxylic acids [29].

Considering the information given above, the generation of GDC can be postulated to occur by the following reactions:

α-d Glucopyranose → Gluconolactone/Gluconic acid + 2e^−^ + 2H^+^ (1)

2Cu^2+^ + 2e^−^ + 2H^+^ → 2Cu^1+^ + 2H^+^ (2)

2Cu^1+^ + 2H_2_O_2_ → 2Cu^2+^ + OH^−^ + OH (3)

2OH + α-d Glucopyranose → Gluconolactone/Gluconic acid + 2H_2_O (4)

Gluconolactone/Gluconic acid + Cu^2+^ ↔ GDC (5)

The synergism in the generation of GDC produced by incubations performed with the mixture glucose/Cu^2+^/H_2_O_2_ can be explained by the occurrence of Reactions (1) and (2), followed by Fenton-type Reaction (3).The oxidation of glucose in the C1 position yielding d-1,4-gluconolactone or in C6 yielding glucuronic acid, has also been found in the glucose oxidation in aqueous colloidal MnO_2_ surface as well as in supported metal catalysts including platinum or palladium [30,31]. It has been recently reported that glucose oxidation mediated by free radicals generated by high-frequency ultrasound generate d-glucoronic acid as the main products [32]. We can discard that GDC can be composed by d-glucoronic acid because of the absence of hemiacetal H signals observed in the ^1^H-NMR experiments. The generation of metal-gluconate complexes has been reported, and many of these complexes present absorption in the UVA-visible region [26,27].

With the aim to determine whether GDC can be associated with oxidative and glycoxidative damage induced in lens proteins during incubation with glucose, mixtures of glucose/Cu^2+^/eye lens proteins were incubated during 10, 20 and 30 days at 37 °C. The analysis of deproteinized solutions of these mixtures depicted an increase in the absorbance at 365 nm, showing a linear behavior with the incubation time. This fact is consistent with a previous report in which mixtures of glucose/Cu^2+^/eye lens proteins presented photosensitizing capacity after 10 days of incubation, which was attributed to GDC generation [7]. The mixture glucose/Cu^2+^/eye lens proteins also showed an increase of hydrogen peroxide concentration with the incubation time (Figure 7B). In addition, an increase in the levels of carboxymethyl-lysine was observed after 20 and 30 days of incubation; a similar behavior has also been observed during long term incubations of glucose with collagen [33]. We have found positive and significant correlations between the absorbance of chromophores generated during the incubation of the mixture glucose/Cu^2+^/eye lens proteins and the levels of hydrogen peroxide and carboxymethyl-lysine. This fact suggests that GDC could be used to determine the contribution of autoxidative reactions during protein glycation induced by glucose, considering that our results demonstrated that GDC was generated by glucose autoxidation.

## 4. Materials and Methods

### 4.1. Reagents

d(+)-Glucose (≥99.5%), Na_2_HPO_4_·7H_2_O, NaH_2_PO_4_·2H_2_O, Xylenol Orange tetrasodium salt and CuSO_4_·5H_2_O were obtained from Sigma-Aldrich (St. Louis, MO, USA). Pierce™ BCA Protein Assay Kit was obtained from Thermo Scientific (Rockford, IL, USA).

Ultrapure water obtained from a Barnstead EasyPure water filter system (Thermo Scientific, Dubuque, IA, USA) was used for the preparation of all solutions. All solutions were filtered through a 0.2 μm sterile nitrocellulose filter.

### 4.2. Isolation of Water-Soluble Bovine Lens Proteins

Bovine lenses were obtained from the slaughter-house. Twenty bovine lenses were decapsulated and stirred in a 50 mM Tris–HCl buffer pH 7.4 containing 0.2 mM KCl, 1 mM EDTA, 10 mM β-mercaptoethanol and 0.05% NaN_3_. The suspension was homogenized and centrifuged at 15,000 rpm for 30 min at 4 °C (Sorvall Superspeed RC2-B). The supernatant was extensively dialyzed against deionized water at 4 °C. Protein concentration was determined by means of the BCA Protein Assay Kit, according to the manufacturer’s instructions. Potassium buffer 0.1 M pH 7.4 was added in order to achieve a protein concentration of 50 mg/mL.

### 4.3. Evaluation of Oxidative Glycoxidative Stress in the Generation of the Major Chromophore Arising from Glucose Degradation

Sterile solutions of glucose (5 mM) were incubated with and without CuSO_4_ (5–50 μM), H_2_O_2_ (0.5–5 mM), glyoxal (0.5–5 mM) or methylglyoxal (0.5–5 mM). Solutions were prepared in phosphate buffer 100 mM, pH 7.4, which was previously treated with Chelex in order to decrease the concentration of metals. Incubations were carried out during 10, 20 and 30 days in triplicate. Samples were prepared in order to analyze them the same day after the incubation period. 

### 4.4. Generation and Purification of the Major Chromophore Arising from Glucose Degradation

Glucose (250 mM) was prepared in phosphate buffer 100 mM, pH 7.4 in sterile conditions in the presence of CuSO_4_ 5 µM. This solution was incubated during 30 days at 37 °C in the dark. After this period, glucose concentration was determined by photometric measurements by means of glucose assay kit (Sigma-Aldrich, St. Louis, MO, USA), according to the manufacturer’s instructions. At the end of this period, the absorption spectra were recorded on a UV-visible photodiode array (PDA) spectrophotometer (Cary 8454, Agilent, Santa Clara, CA, USA). The samples were partially lyophilized, distributed in aliquots and kept frozen at −20 °C until purification. Thawed samples were purified by column chromatography on a Sephadex LH-20 (Pharmacia Fine Chemicals, Piscataway, NJ, USA). Milli Q water was used as the mobile phase. The different fractions were recovered by means of a fraction collector (FC 203B, Gilson, Middleton, WI, USA). The flow rate was adjusted to 0.25 mL min^−1^, collecting in each tube a volume of 1 mL. The fractions were analyzed by means of absorption spectroscopy and determining the glucose concentration by means of a glucose assay kit. Purification was achieved selecting the fractions enriched in the chromophore (λ_max_ = 365 nm) but low in glucose concentration.

### 4.5. Nuclear Magnetic Resonance (NMR) Analyses

The NMR spectra were recorded on a Bruker Avance 400 (Bruker, Rheinstetten, Germany) spectrometer at 400 MHz for ^1^H and 100 MHz for ^13^C in D_2_O. Chemical shifts are given in ppm using residual water for calibration. 

### 4.6. FT-IR

FT-IR spectra were obtained employing a Nicolet Nexus, 470-FT-IR Spectrophotometer (Thermo Scientific, Madison, WI, USA). Glucose and GDC samples were scanned as KBr disks at a concentration of 1% *w*/*w*. The disk was prepared by applying a 40 mPa pressure during 2 min. The scan was performed in the range 700–4000 cm^−1^ at a resolution of 4 cm^−1^ and with 32 scans per spectrum. 

### 4.7. Immunodetection and Quantification of Carboxymethyl-Lysine

SDS-PAGE analysis was carried out boiling 22.5 μg of protein per sample for 5 min in Laemmli sample buffer and loaded into 12% (*w*/*v*) SDS-PAGE gels. Electrophoresis was performed at 100 V for 1–2 h. Proteins were electrotransfered or stained with 0.1% (*w*/*v*) Coomassie Brilliant Blue and washed-out in a solution containing 10% (*v*/*v*) ethanol and 0.75% (*v*/*v*) acetic acid up to 24 h. 

Proteins were electrotransfered onto a Hybond nitrocellulose membrane (GE Healthcare, Saclay, France), which was then blocked with PBS supplemented with 1% BSA and 0.1% Tween 20. Membranes were incubated with an anti-carboxymethyl-lysine monoclonal primary antibody (R&D Systems, Minneapolis, MN, USA) at a dilution of 1/1000 and incubated overnight under agitation at 4 °C. Then membranes were washed with PBS, and incubated with HRP-conjugated secondary antibodies. Membranes were revealed with ECL Plus chemiluminescent detection system (Amersham, GE Healthcare, Buckinghamshire, UK). Blots were scanned and signal intensities were quantified with ImageJ software (NIH, 1.48v, Bethesda, MD, USA). 

### 4.8. Peroxides Quantification

Mixtures of lens protein (10 mg/mL)/glucose (30 mM)/CuSO_4_ (5 μM) and glucose (30 mM)/CuSO_4_ (5 μM) were incubated during 0, 10, 20 and 30 days at 37 °C, pH 7.4 in sterile conditions and peroxide concentrations were determined immediately after the incubation period was completed. Modified FOX-2 method was used for the determination of hydrogen peroxide and protein hydroperoxides. Total peroxides were determined by mixing 300 μL of incubated solutions (samples) with 50 μL of the working reagent and completed at 1 mL with milli-Q water. The working reagent contained 5 mM of ferrous sulfate and 4 mM of Xilenol Orange in 0.5 M H_2_SO_4_. An additional 15 min pre-incubation step with 20 μL of catalase in a concentration of 715 U mL^−1^ (instead of water) was carried out to eliminate H_2_O_2_ and measure only protein hydroperoxides. The mixtures were vortexed and left to react for 30 min in the dark at room temperature. The absorbance was quantified at 560 nm in an UV/vis spectrophotometer (Spectroquant^®^ Pharo 300, Merck, Darmstadt, Germany) and the concentrations were interpolated from a standard curve made with H_2_O_2_. To eliminate the interference of phosphate buffer, hydrogen peroxide standards were prepared considering a volume of 300 μL of 100 mM phosphate buffer, pH 7.4 and a good correlation was found.

### 4.9. Statistical Analyses

Experiments were performed in triplicate and results are expressed as mean values ± SD. Correlations between variables were analyzed by Pearson correlation with a 95% confidence limit.
